# Computer vision-aided bioprinting for bone research

**DOI:** 10.1038/s41413-022-00192-2

**Published:** 2022-02-25

**Authors:** Changxi Liu, Liqiang Wang, Weijie Lu, Jia Liu, Chengliang Yang, Chunhai Fan, Qian Li, Yujin Tang

**Affiliations:** 1 State Key Laboratory of Metal Matrix Composites, School of Material Science and Engineering, Shanghai Jiao Tong University, No. 800 Dongchuan Road, Shanghai, 200240 China; 2 Affiliated Hospital of Youjiang, Medical University for Nationalities, Baise, Guangxi 533000 China; 3 School of Chemistry and Chemical Engineering, National Center for Translational Medicine, Shanghai Jiao Tong University, Shanghai, 200240 China

**Keywords:** Bone, Bone quality and biomechanics

## Abstract

Bioprinting is an emerging additive manufacturing technology that has enormous potential in bone implantation and repair. The insufficient accuracy of the shape of bioprinted parts is a primary clinical barrier that prevents widespread utilization of bioprinting, especially for bone design with high-resolution requirements. During the last five years, the use of computer vision for process control has been widely practiced in the manufacturing field. Computer vision can improve the performance of bioprinting for bone research with respect to various aspects, including accuracy, resolution, and cell survival rate. Hence, computer vision plays a substantial role in addressing the current defect problem in bioprinting for bone research. In this review, recent advances in the application of computer vision in bioprinting for bone research are summarized and categorized into three groups based on different defect types: bone scaffold process control, deep learning, and cell viability models. The collection of printing parameters, data processing, and feedback of bioprinting information, which ultimately improves printing capabilities, are further discussed. We envision that computer vision may offer opportunities to accelerate bioprinting development and provide a new perception for bone research.

## Introduction

Bone tissue engineering is an advanced science that aims to accelerate medical transformation and shorten the distance between scientific research and clinical practice.^1,2^ A critical challenge for bone tissue engineering is to produce three-dimensional (3D) vascularized cellular constructs precisely and repeatedly, with clinically relevant properties such as size, shape and structural integrity.^3^ To address this challenge, bioprinting has provided new perspectives and has shown great promise.

Bioprinting originates from a synthetic human bladder scaffold with patient cells fabricated by Anthony Atala’s team, but this explanation of the onset of bioprinting is controversial since the employed method utilizes a traditional mold manufacturing process.^4^ Bioprinting has been developing rapidly with contributions from Tom Boland,^5^ Garbor Forgacs,^6^ and Douglas Chisey’s team in 2003.^7^ Later, Brian Derby, Doug Chrisey, and Vladimir Mironov defined bioprinting as the utilization of material transfer processes to pattern and assemble biologically relevant materials (such as molecules, cells, tissues, and biodegradable biomaterials) with a prescribed organization to accomplish one or more biological functions.^8^ The prime purpose of bioprinting is to achieve organ transplantation and organ regeneration,^9^ and this purpose has expanded to the exploration of highly biomimetic and reliable in vitro models in high-throughput experiments.^10^ To date, researchers have successfully achieved bioprinting of a human heart.^11^ However, more investigations are required for bioprinting bone scaffolds and cartilage,^12,13^ and the repair of complex organs needs further experimentation.^14^

Bone is among the most commonly transplanted solid tissues,^15^ and thus significant efforts have focused on bone tissue biofabrication. As a powerful biofabrication tool, bioprinting has shown clinical potential in the bone research field, with advantages including high specificity, high precision, and low cost, which are consistent with the characteristics of additive manufacturing (AM).^16,17^ Aided by computer vision, this method can produce bioprinted scaffolds or bioengineered grafts with multiple types of cells and biomaterials, with precise control over shape, size and spatial placement, which would greatly contribute to bone implantation. Computer vision is a simulation of biological vision, and its main task is to identify objects and determine the suitable orientation to achieve rapid positioning, object size measurements, defect detection, and object sorting.^18^

The objective of this review is to discuss recent computer vision-aided bioprinting for bone research. The second chapter mainly introduces the development of bioprinting and computer vision. The third, fourth and fifth chapters mainly present bone scaffold trajectory correction, bone defect detection, and cell viability models in bioprinting during the last five years.

## Bioprinting and computer vision

Bioprinting with acellular and cell-laden bioinks can be categorized into two groups: acellular bioprinting and cell-laden bioprinting. For acellular bioprinting, the mechanical properties of materials, such as high toughness and low sliding friction, are required immediately. Xu et al.^19^ summarized recent high-strength and elastic bioinks with various elaborate structures (double-network and single network) and nanocomposite bioinks that have excellent mechanical properties and stability. In addition, a biocompatibility test is required due to cell reinjection and cell culture for acellular bioprinting. In cell-laden bioprinting, approximately (5–40) × 10^6^ cells per mL cells are typically added to the bioink. Bioink provides an environment for cell survival and growth in printing, and it also supplies strong enough mechanical support for the overall structure.^20^ For cell-laden bioprinting, the final cell viability is as important as the mechanical properties in acellular bioprinting.

Regarding the printing method, bioprinting can be generally divided into four types, i.e., inkjet-based printing, laser-based bioprinting, extrusion-based bioprinting, and stereolithography-based bioprinting.^21^ A summary of these bioprinting methods and their related pros and cons are presented in Table 1. Extrusion-based bioprinting is currently the most widely used method in the bone research field. In this method, a mechanical force is generated by the rotation of a screw or an air pump connected to an extrusion cylinder. Through this mechanical force, the bioink is smoothly extruded from the extrusion cylinder. In the bone printing field, extrusion-based bioprinting has many advantages over other bioprinting methods and is currently considered the most suitable method for printing human cartilage and bone scaffolds due to its high-throughput performance and large volume. In addition, extrusion-based bioprinting can produce high-viscosity bioinks (~600 kPa·s), with cell densities reaching those found in natural tissue.^21^

**Table 1:** Bioprinting technologies and their pros and cons according to ASTM standard F2792^107^

Manufacture	Process	Technology	Benefits	Limitations	Refs
Inkjet-based printing	Materials jetting	Inject printing	High 2D resolution	Lacks the Z direction	^108–114^
	Binder jetting	Powder bed and inkjet head printing (3D powder)	High 2D resolution	Low cell compatible	^115–121^
Laser-based bioprinting	Powder bed fusion	Selective laser sintering	Mechanical strength Faster and higher resolution than other powder methods	Low cell compatible	^122–128^
Extrusion-based bioprinting	Material extrusion	Fused deposition modeling (FDM)	Ultrahigh throughput	Thermoplastic materials only and low resolution	^129–133^
		direct write	Variety of materials	Low resolution and accuracy	^134–140^
Stereolithography-based bioprinting	VAT polymerization	Stereo lithography (SLA)	3D resolution high accuracy	Cell photo-induced damage	^141–147^

Currently, extrusion-based bioprinting has been one of the most widely used bioprinting methods in the bone scaffold printing field; however, its low printing resolution (~100 μm)^22^ remains one limitation. Resolution is a significant parameter that influences the final printing performance of bone scaffolds. For instance, an investigation of the effect of 3D bioprinting on the differentiation and mineral precipitation of bone cells compared to the film area indicated improved results under 3D conditions.^23^ This suggests that bone structure needs higher requirements for lattice structure accuracy and printing process resolution since different morphologies affect osteoblast precursor cell differentiation and mineral precipitation. To improve the bone structure accuracy and increase the resolution, reducing the size of the nozzle diameter was proposed, but this led to increased shear stress values in the nozzle head, which may damage cells.^24^ Therefore, depending on the choice of the nozzle diameter, the final cell viability will vary from 45% to 95%.^25^

**Fig. 1:**
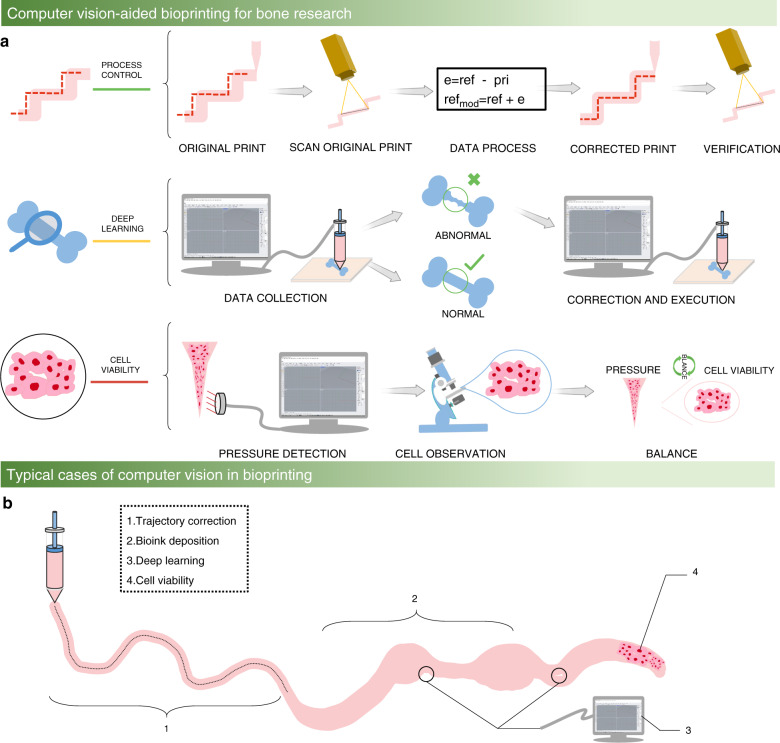
Computer vision-aided bioprinting for bone research. **a** Schematic illustration of computer vision to improve the bioprinting performance for bone research. From top to bottom, the top part illustrates the processes of trajectory detection and correction during the bioprinting process control, which include the original print, scan original print, data process, corrected print, and verification. The middle part shows the application of deep learning in the diagnosis of bone diseases, and the bone disease data are collected and analyzed to determine the condition of the disease. The bottom part illustrates the collection of cell viability under different pressures to determine the balance between pressure and cell viability. **b** Example of typical cases of computer vision optimizing the bioprinting process to improve the final bone bioprinting quality

Computer vision provides new insights to address the problems of low accuracy and low resolution in extrusion-based bioprinting for bone research. During the past ten years, computer vision has developed from simple binary image processing to large-volume data and high-resolution image processing thanks to the development of artificial intelligence, microelectronics, big data, and deep learning.^26^ In addition to image processing, computer vision also includes the collection and processing of other information during the manufacturing process. Figure 1A illustrates the computer vision process, which uses sensors (such as high-speed cameras, thermocouples, pressure sensors and microphones) to collect the information of the entire printing process (such as pictures, temperature, pressure, and sound), which can be stored in a computer. Consequently, the computer is used to calculate and analyze the defects created during the printing process, and then this information can be fed back to the bioprinting algorithm to realize process control. Figure 1B shows how computer vision adds control to different parts of the bioprinting process to achieve trajectory correction, width detection, deep learning, and cell viability detection.

Since most AM techniques have printing defects, including extrusion-based bioprinting, it is necessary to use computer vision and process control to repair defects and correct errors, including biomedical areas,^27,28^ aerospace,^29^ tooling,^30^ and metals.^31^ For instance, it has been observed that bone scaffold failure is often accompanied by local stress concentrations during porous scaffold printing. The utilization of computer vision to collect stress data following the optimization and improvement of the stress concentration state can lead to enhanced scaffold quality.^32^ A nondestructive evaluation by computer vision can be accomplished by collecting a large number of pictures of defects during the printing process.^33^

Furthermore, deep learning has been demonstrated to be a useful method for a wide range of computer vision image tasks. In particular, convolutional neural networks (CNNs) can segment a bone image region of interest. For instance, input radiographs can be standardized and preprocessed, and bone age assessments can be performed.^34^ Since CNNs can automatically recognize the hierarchy of discriminative features by training a set of labeled bone images, this technique can also identify various defects and porosities in the interlayer boundaries during printing,^35^ which play a vital role in bone printing performance analysis.

## Bone printing process control

**Fig. 2:**
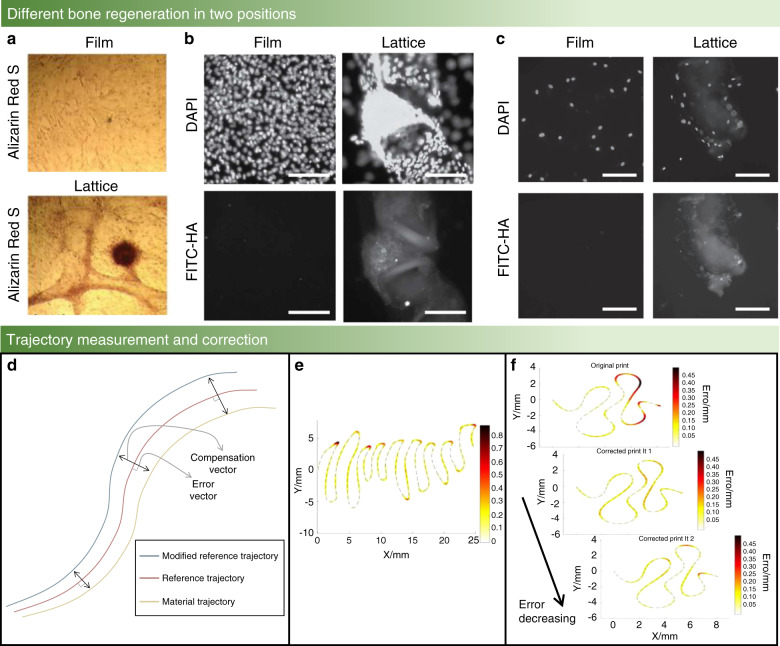
Measurement and correction trajectory of a bone scaffold. **a** Light micrograph shows calcium deposition tests of MG63 cells growing on films (top) compared to growing on GelMA lattices (bottom) under the same conditions (Day 21).^23^ **b** Confocal fluorescent images show mineral deposition by MG63 cells under conditions as in **a**.^23^ **c** Confocal fluorescent images show lattices alone triggering mineral deposition by osteoblasts (NHOst) cells. NHOst cells deposit hydroxyapatite (bottom right panel) and attach to the GelMA lattice (top right panel) and did not stain positively for hydroxyapatite (left panels) on GelMA films.^23^ **d** Example path representing the normal vector approach. **e** Different shape errors are different under the same bioprinting conditions. The shade of the color shows the level of the error, and a darker area means a higher level error; otherwise, a lighter area means a lower level error.^40^ **f** From top to bottom, the plots show the task error for the scaffold error, the first iteration of correction error, and the second iteration of correction error.^40^ (Figures adapted with permission from McBethe et al. ^23^ and Armstrong et al.^40^)

Extrusion-based bioprinting faces the limitation of low printing resolution. The importance of bioprinting resolution has been well explained by Lee et al.,^11^ who also described how to precisely control the accuracy of bioprinting and improve the resolution. These authors suggested that high-resolution guarantees successful bioprinting of heart tissue. McBeth et al.^23^ studied the three-dimensional structure of the differentiation and precipitation of osteoblast precursor cells and observed better differentiation of cells and a larger number of mineral deposits in the lattice structure area than in the film structure area (Fig. 2a–c). Lee and McBeth’s research studies^11,23^ both show that the accuracy and resolution of bioprinting have a substantial impact on the feasibility and quality of printing components, which also play a significant role in bone cell growth, bone cell division, and bone cell differentiation. Resolution is also a very important performance parameter in organ-on-a-chip platforms; this new technology can provide high-throughput screening of candidate drugs against toxicity, with a particular emphasis on bone marrow-on-a-chip.^36^ The specific content of organ-on-a-chip platforms will be discussed later. Miri et al. ^37^ suggested that the success criterion for fabricating organ-on-a-chip platforms is the structural fidelity of the bioprinted microtissues, partly determined by the minimum feature size known as the bioprinting resolution. For these reasons, solving the low-resolution problem is an urgent issue in bone bioprinting.^38,39^

A major limitation of extrusion-based bioprinting is the lack of sensing and direct process control that has resulted in lower resolution than bioprinting under ideal conditions. Two different frames are required when considering motion control for bioprinting, i.e., the machine and extruder axis frame and the material deposition frame. There is a reference trajectory for the joint space frame in bioprinting, which is defined as a set of points for the axes to follow to trace the as-designed shape.^40^ To transform bone bioprinting into a clinical transplantation application platform, an improvement of the printing resolution is necessary, which requires utilization of computer vision to measure and correct errors during the bone bioprinting process.

### Measurement and correction trajectory of a bone scaffold

Generally, trajectory errors can affect the resolution of bone scaffold bioprinting. More trajectory errors lead to lower accuracy and resolution in bioprinting. Therefore, reducing trajectory errors is the primary task to improve the resolution of bone scaffold bioprinting.

To improve accuracy and resolution, researchers have developed multiple algorithms to track and correct scaffold trajectories. The use of a parametric interpolation technique to optimize the machining process, which is a traditional and effective method, was first mentioned in the Computerized Numerical Control Machine machining field.^41^ On the basis of the parametric interpolation technique, the correction method adopts the chord-tracking algorithm (CTA) and predictor-corrector interpolator (PCI) algorithms. First, data acquisition is performed by laser sensor scanning to obtain the 3D point cloud data of the material structure. Next, the 3D point cloud data are analyzed by CTA and PCI on the actual trajectory and reference trajectory to obtain a modified trajectory, and then the test is executed according to the modified trajectory. However, early investigations only identified and analyzed the errors without correcting them, which means that the remaining errors are still present in the entire bone scaffold. For instance, Duan et al.^22^ discovered and evaluated the errors in the printed rectilinear lattice structure and for the first time measured the difference in printing accuracy between the channel dimensions of the material part and the reference part, which used the percentage of the overlap of two parts to indicate the error size. Later, Hockaday’s team^42^ calculated the three-dimensional errors between the geometry of valve conduits and the original stereolithography (STL) model to assess the external geometric fidelity.

Armstrong et al.^40^ measured the two-dimensional trajectory of the repeated bending of bone scaffolds with different degrees and performed multiple corrections during the bioprinting process. The whole trajectory measurement and correction method is illustrated in Fig. 1a. First, the bone scaffold followed the reference trajectory without correction. Then, the bone scaffold shape of the component was scanned to obtain 3D point cloud data using a laser sensor.^43^ Next, the bone scaffold trajectory errors were calculated to obtain a new modified reference trajectory. Finally, the material was printed according to the new modified reference trajectory, and the bone scaffold trajectory errors were measured again.^44^

Bone scaffold trajectory correction can be accomplished with the assistance of MATLAB^45,46^ or Python^47,48^ software. In this regard, the actual bone scaffold trajectory and the reference trajectory coordinates should be put into the same coordinate system. Then, actual bone scaffold trajectory interpolation processing should be performed so that the actual bone scaffold trajectory will have the same vector size as the reference trajectory. Next, a recognized error correction method, introduced by Bandy et al.,^44^ is utilized and the error vector between the actual bone scaffold trajectory and the reference trajectory is identified. Then, the mirroring method is used to modify the reference trajectory so that the modified reference trajectory of the bone scaffold will have the error vector correction, as shown in Fig. 2d.

This series of experiments has led to several conclusions:

1. The error of the bone scaffold trajectory whose shape is close to a straight line is negligible, less than 0.05 mm, but the error at each bend is significant, greater than 0.45 mm (Fig. 2e).
2. The error range depends on the bone scaffold structure size. A smaller print component leads to a higher error range, and a larger print size corresponds to a smaller error range.
3. The effect of the error correction function is different when using two norm and infinity-norm minimization to measure the actual bone scaffold trajectory and reference trajectory; the norm scheme is a mathematical concept used to describe length. The specific results are shown in Table 2.

**Table 2:** The results of the two norm schemes to correct the bone scaffold path^40^

Reference	Two norm			Infinity-norm		
	Origin print	Corrected print	Percent reduction	Origin print	Corrected print	Percent reduction
Scaffold	35.5	24.2	32%	0.9	0.5	49%
4. The error decreases as the number of iterations increases, and the error drops to an acceptable range after the third generation (Fig. 2f).

In another study, trajectory measurements and corrections were performed along with simultaneous adjustment and correction of the bone scaffold width via a method similar to that mentioned above.^49^ The final bone scaffold trajectory error and bone scaffold width error were reduced to an acceptable range (except for the end part). Additionally, the bone scaffold width control problem affects the bioprinting resolution. In the next sections, the bone scaffold width will be further analyzed.

In general, there are some trajectory offset problems in the bioprinting process of bone scaffolds, and these trajectory offset errors will reduce the resolution of bone scaffolds without correction. To improve the accuracy of bone scaffold bioprinting, it is necessary to introduce computer vision to assist in the collection of the errors between the bone scaffold trajectory and reference trajectory; this can be accomplished by using a laser sensor and an algorithm to acquire a new trajectory to eliminate the errors.

### Measurement and correction of the bone scaffold width

For bone implantation, bioink can be directly extruded from the extrusion nozzle during extrusion-based bioprinting due to the force applied by the external air pump or the mechanical force by screw rotation. The extrusion process affects the shape, accuracy, and resolution of the bone scaffold. The pressure of the extrusion nozzle head is a vital bioprinting parameter for bone implantation, and the printed spatial resolution of the bone scaffold changes with fluctuations in the extrusion nozzle head pressure. Most importantly, if the extrusion nozzle head pressure changes too severely and exceeds the tolerance range of bioink, then the bone scaffold will crash during the bioprinting process.^20^ Bellhouse^50^ suggested that the whole geometry of the aortic valve is the key factor to ensure blood flow in tissue engineering. Additionally, Modenesi et al.^51^ found that coronary flow was affected by the geometry of the aortic valve in bioprinting. A previous study on bone replacement implant manufacture by selective laser melting (SLM) was carried out by Egan et al.,^52^ who explored the effect of various energy inputs (33%, 66%, 100%) during the printing process on the resultant mechanical properties. Furthermore, Egan et al.^53^ set three test bone implantation types, namely, (1) control samples, (2) an unintentionally defective sample, and (3) intentionally defective samples, to identify defective layers within the L-PBF process. These investigations provided a new vision for the in situ detection of the printing process.

The restricted application of bioprinting for bone implantation is related to its low resolution in the spatial material structure. An accurate scaffold width is momentous for in vivo investigations, such as in enzymatically cross-linkable materials,^54^ neuron regeneration and connection,^55^ and cell delivery.^56^ This example suggests why precise control of the bone scaffold width plays a vital role in macroscopic organ function as well as in microscopic cell differentiation and growth.

Bone scaffold width is a main factor that affects the resolution of bioprinting; however, to date, measurement and correction studies of bone scaffold widths have been lacking in bioprinting research. The main reason for low resolution is related to the absence of width control of the bone scaffold, which can lead to the overlap of two direction lines, undesirable coverage of designed pores, and overlap of corners. Computer vision not only solves the process control problem for extrusion nozzles but also corrects errors and predicts possible defects. Computer vision shows great promise in the collection of input information, and the establishment of the mapping relationship between input information and output components in both welding and AM seems to be a promising scheme to be followed in bioprinting.

**Fig. 3:**
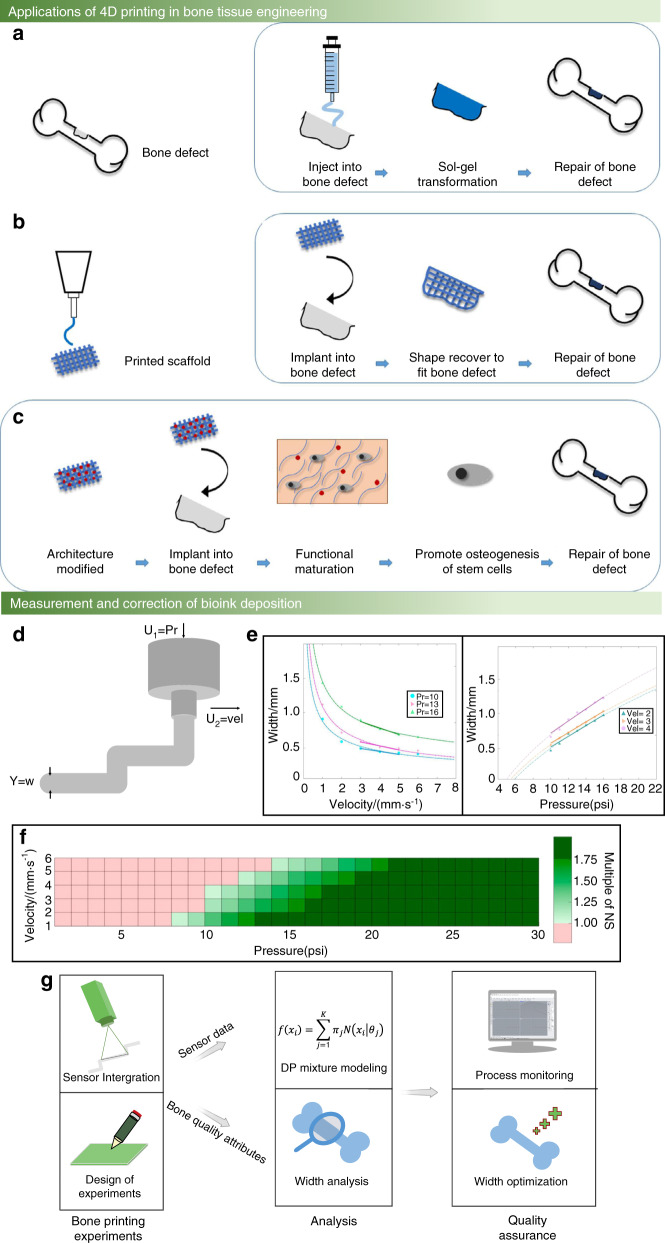
Measurement and correction of the bone scaffold width. **a** Examples of injectable thermosensitive hydrogels for 4D bone tissue regeneration.^57^ **b** Examples of the shape memory of 4D printing to repair bone tissue defects.^57^ **c** Bone tissue repair based on the establishment of a biomimetic microenvironment by 4D printing, which induces the functional maturation of neobone tissue and promotes the osteogenesis of stem cells, enhancing the formation of new bone tissue.^57^ **d** Two factors affecting the bioprinting width (w): input pressure (U1) and printing axis moving speed (U2). **e** The mapping relationship between pressure and width and the mapping relationship between moving speed and width.^59^ **f** The printing width under the combined influence of the extrusion pressure and moving speed. The red area represents a width smaller than the normal width, the dark green area on the right represents a width larger than the normal width, and the middle light green represents a width within the normal range.^59^ **g** Summary of the research approach integrating experimental tests, sensor data, and Dirichlet process modeling for monitoring width control. (Figures adapted with permission from Wan et al.^57^ and Armstrong et al.^59^)

The bone scaffold width is affected by machine parameters and material design. Usually, some specific polymers are designed in the bioink to ensure the structural strength and the cross-linking between the polymers and polymer functional groups; unfortunately, this will affect the bone scaffold width. In particular, some specially designed bone scaffolds have shape memory features that change their appearance under the stimulation of different external environments; this process is called 4D printing.^57^ The bone scaffold ingredient is constant during 4D printing; hence, the bone scaffold width can be altered just by changing the print parameters (Fig. 3a–c). Some scholars use the bioink flow model to predict the bone scaffold linewidth under different nozzle moving speed conditions.^58^ However, this flow model can only predict the overall bone scaffold line width. As a consequence, the performance of the flow model is unacceptable during actual bioprinting, for instance, in the case of bone scaffold linewidth fluctuations caused by the printing pattern.

To mitigate the limitations of the flow model, the correction process can be divided into a measurement part and a correction part, which compensates for the defects of the flow model. Even under the same machine parameters and material parameter conditions, the different pattern shapes can affect the bone scaffold line width. Hence, the measurement part is required to collect the bioink line width by using a camera or laser sensor of different pattern shapes during the printing process. The correction part modifies the bone scaffold line width by altering the mechanical parameters. Additionally, the printing axis moving speed and the nozzle head pressure are two main factors affecting bone scaffold width, as shown in Fig. 3d. Experimental results have shown that very fast movement of the extrusion nozzle in the bioprinting process can lead to instability. For this reason, a preferred approach is to adjust the nozzle pressure changes to control the bone scaffold width. The correction part adjusts the nozzle pressure to achieve the measurement and correction of the bone scaffold width based on the error information collected from the measurement part,^59^ as illustrated in Fig. 3e, f.

Width control of bone scaffolds is also an important way to improve the resolution of bone bioprinting, which is similar to the bone scaffold trajectory correction. Figure 3G summarizes the monitoring research approach for width control. In bone scaffold bioprinting processes, a major challenge is to achieve in situ measurement and correction simultaneously. Currently, both measurement and correction processes are being developed, but simultaneous measurement and correction needs more effort.

## Deep learning for bone research

**Fig. 4:**
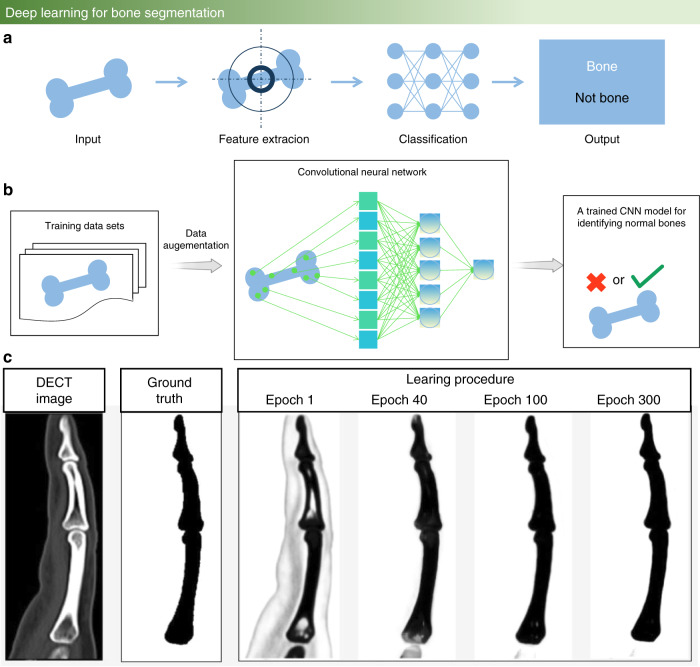
Deep learning for bone research. **a** Overview of the machine learning process. **b** The process of CNN algorithm training to process images and extract features. **c** Optimization of the CNN algorithm for forefinger bone segmentation for calcinosis cutis quantification.^71^ (Figures adapted with permission from Chandrasekaran et al.^71^)

During the last five years, with the rapid development of artificial intelligence, deep learning has been widely employed to achieve clustering analysis of images, as illustrated in Fig. 4a. Deep learning is a constantly iterative and abstract process that constructs many hidden machine learning models and training models to obtain more useful features and improve the accuracy of classification and prediction. Based on powerful feature extraction capabilities, machine learning has been widely used in the bone research field to segment and enhance bone images for medical diagnosis and to implement visual identification.^60^ In addition to visual identification and image enhancement, analyzing bioprinting defects of bone implantation detection is a promising direction scheme for deep learning.^61^

### Convolutional neural network for bone research

A CNN is a common machine learning model that includes weight sharing and local perception technology with two characteristics.^62–64^ Weight sharing: Each neuron parameter is set to be the same, which means that each neuron uses the same convolution kernel to convolute the image. Local perception: Each neuron needs to perceive the local area rather than perceive the global image. These neurons that perceive different parts are combined to obtain global information at a high level. Owing to these two characteristics, CNNs have the following advantages in the bone research field: (1) These networks avoid explicit feature extraction but implicitly learn from the training data. (2) The weights of neurons on the same feature mapping surface are identical, and each network can learn in parallel and reduce the complexity of the network. (3) These networks have a unique advantage in image processing because the input information is well matched with the network topology. Figure 4B shows the process of CNN algorithm training to process images.

The applications of CNNs in the bone research field include bone age assessment, precise calcinosis cutis quantification, and periodontal bone loss classification. Bone age assessment is usually used to assess the difference between the chronological age and biological age of the bones of children, which assists in the diagnosis of child growth,^65^ endocrinology problems and genetic disorders.^66^ Bone age artificial assessment is usually used to observe the morphology of the hand bones, and the method of judging ages through features matches the characteristics of the CNN. Research studies have collected a total of 8 325 radiographs with age labels diagnosed by skeletal doctors, including 4 278 radiographs for females and 4 047 radiographs for males. Using 70% of the total radiographs as the training set to train the CNN model, the remaining 15% of the total radiographs are used as the validation set to adjust the subsequent CNN model parameters, and the final 15% of the total radiographs are used as the test set to verify the accuracy of the CNN model for bone age assessment. The final bone age assessment results of the CNN model reached 57.32% for females and 61.40% for males.^67^ On this basis, the CNN model can be further optimized. For instance, using five convolutional layers can reduce the training set of bone age assessment to 1 400 radiographs, and the accuracy barely fluctuates.^68^ Using all hand radiographs as the training set, compared to carpal bones or metacarpal and proximal phalange radiographs, the age assessment error will be reduced by 10%–15%.^69^

Although research on the treatment of calcinosis cutis has made great progress, there is still a lack of standardized and validated methods to quantify calcinosis cutis. The subtraction of normal phalanges from segmented bone images is the core step of CNN to quantify residual calcinosis cutis lesions, which uses the mean-shift segmentation algorithm.^70^ The calcinosis cutis quantification of the CNN and expert radiologist measurements are highly consistent (in Fig. 4c).^71^

Statistics of periodic bone loss are an important part of the evaluation and treatment of periodontal diseases. Previously, statistics of periodic bone loss were often provided by clinical evaluations, which were limited by the probing force, angulation, placement, and tip diameter.^72–74^ To date, CNN has been used to identify periodontal diseases by training labeled radiographic tooth images, and the test result quality is similar to that of experienced dentists detecting periodic bone loss.^75^ In addition, the digital results of CNN tests can be displayed in radiographic images, which contain radiographic bone loss information, which is helpful for dentists to diagnose periodontal diseases.^76^

### Deep learning for organ-on-a-chip platforms

Thus far, organ-on-a-chip platforms have made new progress in bone research, including bone regeneration, bone vasculature and innervation, and cancer metastasis to bone. Bioprinting potentially offers tremendous throughput and addresses reproducibility issues faced by traditional organ-on-a-chip systems. In addition, bioprinting embedded into the organ-on-a-chip system allows fabrication of complex in vitro models for mechanistic, simulation, and pharmacological modulation. Deep learning is capable of analyzing a large amount of information obtained by organ-on-a-chip platforms in bone research.

Bone regeneration is a branch of regenerative medicine strategies that consists of bone induction and conduction and involves a number of cell types and intracellular and extracellular molecular signaling pathways.^77,78^ To explore the influence of antibiotics on bone regeneration, inkjet-bioprinting of antibiotic and calcium-eluting micropatterns are used to test high-throughput research and show the advantages of antibiotics with regard to bacterial killing without damaging osteogenic tissue development.^79^

Bone is also a highly vascularized tissue with 10%–15% of the total cardiac circulatory output.^80^ Focusing on bones as a component of the circulatory system, blood vessels in bone are responsible for exchanging oxygen, nutrients, and waste and for providing the hormones, growth factors, and neurotransmitters necessary for the survival of bone cells. Organ-on-a-chip systems can reconstruct the research platform of 3D microvascular networks.^81–84^ In the past, the final quality assessment of drug-aided vascularization was performed by manually analyzing each image before and after drug application. This kind of quality assessment was based on the doctor’s experience by judging whether the generated blood vessel was mature, which relied on human subjective judgment. In addition, there is a low throughput of quality evaluations due to the insufficient number of manual judgments.^85^ After training a large number of vascularized images, the CNN classifies a large number of vascular networks into no-hit, soft-hit, and hard-hit networks. It is the first step in achieving automated drug screening, which is more accurate than manual classification.^86^

Compared with other bone-on-chip studies, bone cancer metastasis is a relatively new research direction, which is a complex multistage process. Some research stimulated MLO-Y4 osteocytes with 1 Pa and 1 Hz fluid for 2 h to investigate the effects of mechanically stimulated osteocytes on breast cancer extravasation.^87,88^ For the discovery of hidden information with regard to in vitro cancer drug treatments, deep learning provides new perspectives based on its big data processing capabilities. For instance, focusing on 3D biomimetic gels of immune cells cocultured with breast cancer cells in organ-on-chip devices, upon treatment with an immunotherapy drug, deep learning classifies cell trajectories very accurately according to the presence or absence of drugs, which proves the existence of cell movement characteristics.^89^

In general, deep learning in computer vision, especially CNN, has been playing an increasingly important role in image processing for bone research and deep information mining. This technology not only helps doctors diagnose specific bone disease pictures through feature extraction of the area of the diseased bone but also includes mining the high-throughput data provided by bioprinting to obtain more deep bone research data.

## Bone cell viability

Biomaterials, cells, and blood supply channels are the three prime challenges in clinical application and regenerative medicine, including the bone bioprinting field. Well-known cellular challenges include cell growth, cell viability and differentiation. Therefore, the final cell viability situation is one of the significant indicators for evaluating cell printing performance. Cell viability is considered a crucial factor during the bone tissue printing process.

**Fig. 5:**
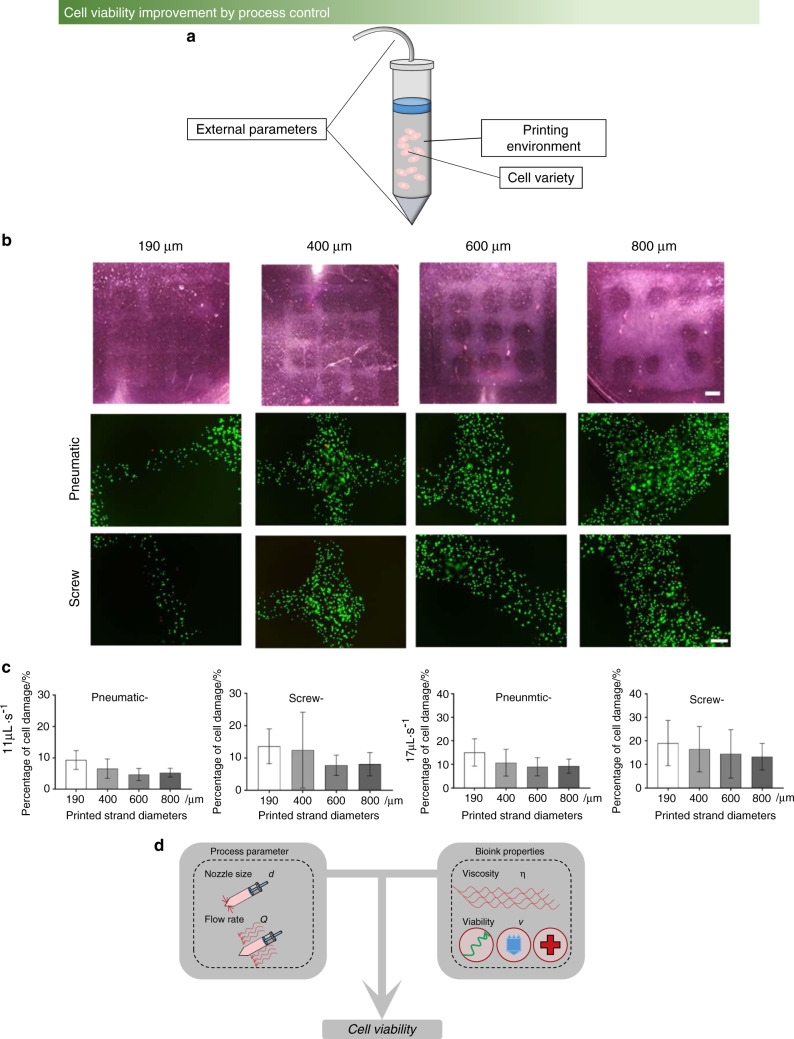
The factor influencing cell viability. **a** The three reasons affecting cell viability and differentiation. **b** Live/dead assay of cells with two pressure methods (pneumatic and screw) at different printing speeds.^92^ **c** Cell damage tests of four different diameters.^92^ **d** Two factors, process parameters and bioink properties, affect cell viability; process parameters, including nozzle size and flow rate; and bioink properties, including viscosity and viability. (Figures adapted with permission from Ning et al.^92^)

The main factors affecting cell viability and differentiation can be mainly divided into three groups, i.e., cell variety, printing environment,^90,91^ and external parameters,^92–94^ as shown in Fig. 5a. First, the survival rate in vitro is different for various cells according to their characteristics and limitations. For example, the survival rates of bone marrow cells, osteoblasts, and chondrocytes in vitro culture are different. Next, the bioink is designed with suitable viscosity and nutrition for cell survival, which provides the required energy for cell growth. Current research is dedicated to establishing a decellularized extracellular matrix (dECM) to improve the survival rate of cells.^95,96^ Finally, unlike other regenerative medicine-based methods, pressure control occurs at the nozzle head, which is a unique part of bioprinting. For instance, there are two different cell viabilities in the two pressure methods, as shown in Fig. 5b, c. The influence of process parameters and bioink properties on cell viability is shown in Fig. 5d.

In the bone research field, compared with osteoblast culturing in bioink without a printing process, the extrusion process during bioprinting may cause osteoblast damage. Most importantly, once the nozzle head pressure exceeds the acceptable range, the final osteoblast viability is decreased. Additionally, the shear force generated in a deposition can cause osteoblast damage during bioprinting. Therefore, selecting appropriate bioprinting parameters is a vital part of the printing process to improve osteoblast viability without replacing the cell variety and modifying the bioink microstructure. However, there is a lack of research reports on pressure detection and control in recent bioprinting studies, and thus, it is urgent to establish the mapping relationship between printing parameters and cell viability.

### Printing parameters and cell viability

**Fig. 6:**
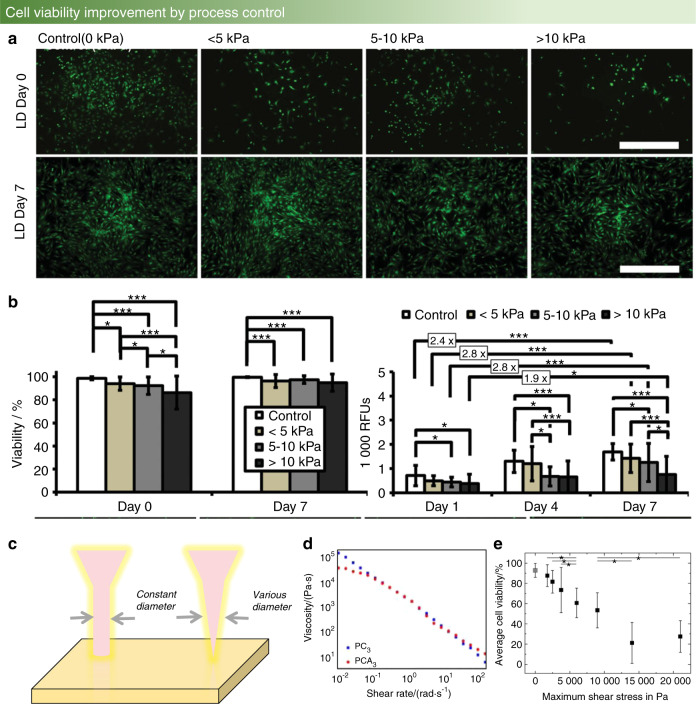
Cell viability in different fabrication environments. **a**, **b** Short-term and long-term impact of different bioprinting-induced shear stress levels on human mesenchymal stem cell (MSC) viability and proliferation.^93^ **c** Cylinder-shaped nozzle head (left) and conical-shaped nozzle head (right). **d** Shear-thinning tests of PCL-PEG-PCL (blue, square) and PCLA-PEG-PCLA (red, circle).^101^ **e** Viability of immortalized mesenchymal stem cells after extrusion-based bioprinting at a concentration of 0.5×10^6^ cells per mL in an alginate-gelatin hydrogel. Maximum shear stress values between 1 747 Pa and 21 000 Pa were generated during printing.^103^ (Figures adapted with permission from Blaeser et al.,^93^ Cui et al.,^101^ and Koch et al.^103^)

Owing to the multifunctionality and wide applications, computer vision can be used to establish the mapping relationship between printing parameters and osteoblast viability. First, an adjustment of the printer nozzle pressure is required, which is influenced by the nozzle diameter and output pressure. Additionally, with a reduction in nozzle diameter, the pressure increases. The output pressure value is calculated by substituting the input voltage value into the established voltage-pressure model. For signal denoising processing and background removal during model building, investigations of Armstrong’s team^38^ can be referenced. Next, the cell survival rate should be counted, which usually uses the TUNEL (TdT-mediated dUTP nick-end labeling) model for cell viability detection. For instance, Fig. 6a, b shows the cell viability under different pressure conditions.

Recently, Ouyang^97^ found that different printing parameters should be used for bioprinting of different cell types to ensure that the printing environment is satisfactory for cell survival, even in the same alginate-gelatin hydrogel bioink. In addition, Pössl et al.^98^ provided a new research vision regarding the shape of the extrusion nozzle, proving that different nozzle shapes can lead to various shear forces. For example, compared with the cylinder shape in Fig. 6c, in which the diameter is constant, the diameter of the conical shape linearly decreases with length, which changes the shear force at different positions and leads to altered cell viability.

Interestingly, it is observed that there is a range of cell survival rates that increases with increasing shear force, rather than linear monotonic changes that decrease with increasing shear force. One explanation is that the intracellular calcium concentration increases under the action of moderate shear stress, which leads to cell proliferation.^93^ For this reason, establishing an acceptable model that also considers the two different impacts of shear force on cell viability is the current challenge of bioprinting.

At present, nozzle pressure control still has many challenges in the extrusion-based bioprinting field. Specifically, when the only aim is to improve bone cell viability, more methods can be used during printing, such as reducing the material strength and output pressure and increasing the nozzle size. However, these methods may lead to a significant decrease in the printability of bioprinting, i.e., insufficient strength, unstable shape, low resolution, and easy collapse. For this reason, the current solution is to utilize the shear force as the threshold for a cell survival rate of 90% to subsequently improve the printability.

### Bioink and cell viability

There is a contradiction between bioink strength and osteoblast viability in bioprinting. High-strength bioink may decrease the cell survival rate, leading to reduced cell viability. Additionally, the insufficient bioink strength for increasing the cell survival rate may cause the collapse of the printing structure. A high flow rate and high viscosity of bioink during bioprinting will generate high shear stresses, which will influence osteoblast viability, proliferation rate, and phenotype.^93–99^ Bioink materials with very high viscosity may reduce the diffusion of nutrition in embedded cells, which is another reason for the contradiction between the viscosity of bioink materials and cell viability.^100^

In a study using a polycaprolactone-polyethylene glycol-polycaprolactone (PCL-PEG-PCL) triblock polymer to enhance the strength, the copolymer showed non-Newtonian properties. This means that the final cell viability performs well due to the reduction in viscosity with increasing shear rate^101^ (in Fig. 6d). Similar results were reported in a study of a dECM bioink in which the cell survival rate was also maintained above 90% by increasing the shear rate and reducing the bioink viscosity.^96^ This research was focused on how to design high-strength bioink, and the measurement of viscosity stays at the data collection level, without an establishment of the mapping relationship between printing parameters and cell survival rates. Furthermore, in the construction of osteochondral tissue in a PCL environment, the survival rates of osteoblasts and chondrocytes under different temperatures and pressure conditions during printing were high. Under conditions of 80 °C and a pneumatic pressure of 400 kPa, osteoblasts and chondrocytes have the highest survival rate.^102^ Koch^103^ studied alginate-gelatin hydrogel bioink, which is a common bioink material in bioprinting. These researchers improved the cell survival rate, without modifying the ingredients of the bioink materials, by adjusting the nozzle diameter and changing the nozzle shape and flow rate to control the pressure, as shown in Fig. 6e. This is consistent with the abovementioned width control results. Furthermore, the cell survival rate decreases to 60% when the stress value is between 5 595 and 10 000 Pa, which is a 30% drop compared with the 90% cell survival rate at 4 078 Pa.

## Summary and future perspectives

The goal of bioprinting is not only to establish tissue models for drug screening and disease modeling in vitro^104^ but also to provide specific functional tissue repair and remanufacturing for in vivo implantation.^21,105,106^ However, bioprinting research is currently at the initial stage, and numerous problems need to be solved with the guidance of multiple and different schemes. To date, in the bone bioprinting research area, many studies have been conducted to improve the structural strength of bone scaffolds by developing and modifying bioinks. However, a systematic accurate collection of information and process control during the printing process is still needed. With the development of computer science, the use of computer vision to achieve process control has attracted much attention in the manufacturing field. With the assistance of computer vision in printing parameter optimization and printing process control, the final structural and biological performance of bioprinted bone scaffolds can be significantly improved.

This review summarizes the recent utilization of the computer vision process in bone research to achieve bioprinting process control, segmentation and enhancement of bone images, and cell viability improvement for bone tissue construction. First, computer vision realizes bone bioprinting process control, mainly including bone scaffold trajectory measurement and correction and bone scaffold width control; this process reduces these kinds of errors in bone fabrication to improve bioprinting resolution. Second, deep learning, especially CNN, has proven to be a powerful method for a wide range of computer vision image tasks. We summarized the utilization of CNN for segmentation and enhancement of the images of bones and bone tissues in medical diagnosis, as well as big data mining and processing of organ-on-a-chip systems. Finally, considering that cell viability is a unique aspect of bioprinting compared with other AM methods, it is necessary to use computer vision to collect the shear force values at the nozzle head, establish the mapping relationship between the shear force and the final osteoblast survival rate, and then locate the printing parameters according to the highest osteoblast survival rate as the threshold.

The ultimate goal of bone bioprinting is that cells can be directly printed and then replaced with diseased bone tissue in the patient body. There is still a long way to go between the current applications and the goal. To achieve this goal, the high accuracy of bioprinting and great cell viability after bioprinting are the main challenges. The following research challenges are put forward to make bioprinting more prosperous for bone research. Many of the studies on current trajectory error evaluation are divided into two steps: detection and correction. Studies of in situ evaluation are lacking at present and are an evolvable area to focus on in the future. Next, to ensure the high accuracy of image recognition for bone diseases, advanced image algorithms proposed in the computer science field might be invested in the application of bone disease diagnosis. Additionally, although the relationship between cell viability and bioprinting parameters has been studied recently,^91,92^ databases between the cell viability of different bioinks and different printing parameters are still lacking at present and should be established as soon as possible to determine the optimal bioprinting parameters. In the future, by further exploring computer vision-aided data collection and printing process control, printing performance and bioprintability will be significantly improved both in vitro and in vivo, which will contribute to the development of bone tissue engineering and translational medicine.
